# Increased Interleukin-6 Activity Associated with Painful Chemotherapy-Induced Peripheral Neuropathy in Women after Breast Cancer Treatment

**DOI:** 10.1155/2010/281531

**Published:** 2010-08-10

**Authors:** Angela Starkweather

**Affiliations:** School of Nursing, Virginia Commonwealth University, Richmond, VA 23298-0567, USA

## Abstract

Accumulating evidence suggests that neural-immune interactions are involved in the development of painful chemotherapy-induced peripheral neuropathy, particularly through the increased release of proinflammatory cytokines. The purpose of this study was used to evaluate levels of interleukin [IL]-6 and IL-6 receptors in women with breast cancer after the conclusion of chemotherapy who either had painful symptoms of chemotherapy-induced peripheral neuropathy (CIPN group, *N* = 20) or did not experience CIPN symptoms (Comparison group, *N* = 20). CIPN participants had significantly higher levels of IL-6 and soluble IL-6R (sIL-6R) compared to women without CIPN symptoms (*P* < .001 for both). In addition, soluble gp130, which blocks the IL-6/sIL-6R complex from binding to gp130 within the cellular membrane, was significantly lower (*P* < .01). Circulating concentrations of sIL-6R were inversely correlated with the density of IL-6R on the cell surface of monocytes in the total sample (*r* = −.614, *P* = .005). These findings suggest that IL-6 transsignaling may be an important biological mechanism associated with the persistence of painful CIPN symptoms, with potential implications for symptom management and research.

## 1. Introduction

Chemotherapy-induced peripheral neuropathy (CIPN) can be a debilitating and often painful consequence of cancer treatment [1–4]. It is estimated that 30–40% of cancer patients experience CIPN, with the incidence varying based on the chemotherapeutic agent used, treatment intensity, including dose and duration of administration, cumulative dose, overall duration of therapy, and coadministration of multiple agents [5].

Chemotherapeutic agents most often associated with CIPN include the platinum-based compounds cisplatin, carboplatin, and oxaliplatin; plant alkaloids vincristine and vinblastine; taxanes such as paclitaxel and docetaxel; eopothilones such as ixabepalone; other agents including thalidomide, lenolidamide, and bortezomib [4]. The precise mechanism of neuronal injury is thought to vary by agent [6]. For instance, plant alkaloids and taxanes cause direct axonal injury and demyelinization by blocking tubulin polymerization, which leads to impaired axoplasmic transport due to microtubule clumping. In contrast, platinum analogs reduce axonal transport and cause apoptosis of dorsal root ganglion cells [7, 8].

CIPN can include alterations in sensory, motor, and/or autonomic function [9]. Sensory changes can include numbness, tingling, hyperesthesia, loss of vibratory perception, and burning pain. Accumulating evidence suggests that inflammatory activation modulated through the increased release of proinflammatory cytokines is a key biological mechanism associated with painful neuropathies [10–12]. 

### 1.1. Chemotherapy-Induced Peripheral Neuropathy in Women with Breast Cancer

Women with breast cancer are often exposed to chemotherapeutic agents that can cause symptoms of CIPN [5]. For most women, painful CIPN symptoms initially manifest during treatment and subside after the cessation of chemotherapeutic agents [6]. However, approximately 15–20% of women with BCA will experience persistent painful CIPN, which is of particular importance in this patient population who are typically young and constitute the largest group of cancer survivors in the United States [13]. Multiples studies have shown that while women with BCA perceive benefit from their cancer treatment, they report problems with persistent painful CIPN, functioning, and global quality of life (QOL) [14–16]. Thus, identifying the factors that influence CIPN symptoms and QOL is of particular importance in this patient population.

### 1.2. Biological Factors Implicated in Chemotherapy-Induced Peripheral Neuropathy

When peripheral nerve damage occurs due to the exposure of neurotoxic chemicals, circulating immune cells, as well as resident immune cells of the nerve fibers, begin to release proinflammatory cytokines into the area of injury [17]. As blood-borne immune cells infiltrate into the damaged region, functional changes occur such as endoneural swelling and breakdown of the blood-nerve barrier [18], allowing direct exposure of neural tissue to inflammatory mediators. Elevated levels of proinflammatory cytokines such as interleukin [IL]-1, IL-6, and tumor necrosis factor [TNF]-alpha are found after nerve injury, and neuropathic pain is attenuated by suppressing the release of these molecules [19–21]. 

IL-6 in particular has been shown to play a large role in the inflammatory process following nerve injury and has been implicated in the initiation and maintenance of neuropathic pain [22–24]. However, IL-6 activity is dependent upon the distribution of receptors on specific cell types to which it can bind. The distribution of membrane-bound (IL-6R) receptors, to which IL-6 can bind directly, is fairly limited throughout the body, existing mainly on hepatocytes and certain subsets of leukocytes. In contrast, IL-6 can complex with soluble receptor IL-6R (sIL-6R) to activate the signal transducing receptor, gp130, which is expressed nearly ubiquitously among all cell types [25, 26]. This means that cells capable of responding to IL-6 alone are restricted to IL-6R + cells whereas virtually all cells respond to the IL-6/sIL-6R complex. Unlike other soluble cytokine receptors that inhibit cytokine signaling, such as those for TNF-alpha, sIL-6R prolongs the half life of IL-6 and amplifies its inflammatory actions by allowing gp130 + cells to respond to IL-6 [27, 28]. Soluble gp130 (sgp130) can inhibit IL-6 activity by binding to the sIL-6R/IL-6 complex thereby preventing its attachment with gp130 within the cellular membrane. Thus, a higher number of sIL6-R receptors could increase IL-6 activity, thereby increasing inflammation and sensitization of the peripheral nerves, whereas higher sgp130 levels could impair IL-6 activity. 

It was recently reported that women with breast cancer (BCA) who have symptoms of CIPN are three times more likely to suffer from persistent neuropathic pain compared to women who do not experience CIPN [29]. Activation of the inflammatory cascade may represent a significant factor that determines the presence of painful CIPN symptoms and the transition to neuropathic pain. The identification of specific biological mechanisms involved in the manifestation of painful CIPN and reduced QOL may provide a means to monitor for those who may be at risk before symptoms occur and QOL declines and may provide a potential therapeutic target to prevent or treat persistent CIPN symptoms. Therefore, the primary aim of this study was to examine the levels of IL-6 and IL-6 receptors (IL-6R and sIL-6R) in women who reported either painful CIPN symptoms or no CIPN symptoms following BCA treatment. The secondary aim was to explore the relationships among biological factors and health-related QOL.

## 2. Materials and Methods

A two-group comparison design was used to evaluate IL-6 and IL-6 receptors in women with BCA who either reported painful symptoms of CIPN or who had no symptoms of CIPN after chemotherapy. This study was approved by the Institutional Review Board residing over the recruitment sites and written informed consent was obtained from all participants.

### 2.1. Setting and Sample

Recruitment took place at three oncology clinics located in the northwest region of the United States. Fliers about the study were posted at each study site, which asked potential participants with painful CIPN symptoms to contact the Primary Investigator (PI) if they were interested in learning about the study. In addition, research nurses at each site approached potential participants about the study, and if interested, they were contacted by the PI. Women diagnosed with BCA were eligible for participation if they met the following criteria: (1) theywere between 6 and 12 months after diagnosis of grade II-III BCA and had completed systemic adjuvant cancer therapy, (2) had no history of other cancer, diabetes, cardiovascular disease, psychological disorder (major depression, anxiety, schizophrenia, or bipolar disorder), immune-based comorbidity (HIV, lupus, and multiple sclerosis), renal disease, alcoholism, or peripheral neuropathy before the administration of systemic chemotherapy, (3) absence of current or recent infection, immunization, hypothyroidism, or vitamin deficiency, (4) and were proficient in English.

### 2.2. Materials

#### 2.2.1. Demographics

Demographic information consisted of interval, categorical, and continuous variables, including: age, race, ethnicity, marital status, level of education, past medical history, medication prescribed and OTC usage, and socioeconomic status. 

#### 2.2.2. Pain

The Short-Form McGill Pain Questionnaire (MPQ-SF) is a well-established self-report measure that entails fifteen verbal descriptors of sensory and affective dimensions of pain, a present pain intensity scale, and the evaluative overall intensity of total pain experience scale [30]. Subjects are asked to rate the verbal descriptors (none-mild-moderate-severe) and items are summed to provide the total pain rating index (score ranges from 0 to 45). Present pain intensity is measured by a 100-mm visual analog scale (VAS); patients are asked to place an “X” on the line from “no pain” to “worst possible pain”. The overall intensity scale is categorical ((0) no pain; (1) mild; (2) discomforting; (3) distressing; (4) horrible; (5) excruciating). Patients are asked to place a checkmark next to the descriptor that reflects their overall intensity of the total pain experience. Reliability of the MPQ is good and is reported to be between 0.64 and 0.87 [31].

#### 2.2.3. Quality of Life

 QOL was measured with the Medical Outcomes Short-Form (SF-36) version 2. The SF-36 is the most widely used health-related QOL instrument in the world [27]. The SF-36 measures eight health concepts: (1) limitations in physical activities because of health problems; (2) limitations in social activities because of physical or emotional problems; (3) limitations in usual role activities because of physical health problems; (4) bodily pain; (5) general mental health (psychological distress and well-being); (6) limitations in usual role activities because of emotional problems; (7) vitality (energy and fatigue); (8) general health perceptions. All items are scored using Likert scales so that a higher score reflects a more favorable health outcome. The SF-36 is scored so that all scales are on the same metric, where 50 is the mean for the US general population and 10 is the standard deviation. Norm-based scoring equates all scores, so scores above 50 are better than the general population average for all scales, while scores below 50 are worse. Test-retest reliability of the SF-36 is reported to be high (0.85) while internal consistency (*α* = 0.88 and 0.95) is good [31].

#### 2.2.4. IL-6 and IL-6 Receptor Measurements

 Serum samples were separated according to standard procedures and stored at −70°C for batch testing. Serum levels of IL-6, soluble IL-6R, and soluble gp130 were measured in triplicate using Quantikine High Sensitivity Immunoassay kits (R&D Systems, Minneapolis, MN). Analyte capture was carried out in 100 *μ*L of serum incubated 2 hours at room temperature with constant shaking. The plates were then washed and incubated with conjugate antibody 2 hours at room temperature. The plates were washed again and incubated with substrate for 1 hour, amplifier for 30 minutes, and stop solution. All laboratory procedures and quality control measures were carried out according to the manufacturer's instructions. The intra-assay precision of all tests was less than 8%; while interassay precision was less than 9%. IL-6 sensitivity = 0.04 pg/ml; sIL-6R sensitivity = 6.5 pg/mL; sgp130 sensitivity = 0.08 ng/mL. 

When circulating sIL-6R levels rise, there is a decrease in the expression of cellular CD126 (IL-6R) due to cytokine-induced receptor shedding [32]. In order to examine the association between receptor shedding [of IL-6R] and sIL-6R levels, the density of IL-6R on monocytes (CD3^−^/CD126^+^/CD14^+^) was analyzed. This cell type was selected because monocytes remain elevated for a prolonged period of time after immune activation as opposed to neutrophils and lymphocytes, which are quickly cleared away and exhibit a less robust response to increased IL-6 levels [32]. Fluorescence-conjugated antibodies were used to identify IL-6R receptors (R&D Systems, Minneapolis, MN). Blood specimens were collected in heparinized vacutainers and Ficoll (R&D Systems, Minneapolis, MN) density gradient was used to separate peripheral blood mononuclear cells (PBMCs). Following this, PBMCs were cultured in RPMI 1640 +10% fetal bovine serum at 37°C in 5%  CO_2_. PBMCs were stimulated with 10 ng/mL of IL-6, and IL-6R cell surface expression was assessed 12 hours later. Cells were analyzed by flow cytometry (FACScan, BD Immunocytometry, San Jose, CA) using FITC-conjugated anti-CD3 to exclude T cells, phycoerythrin-conjugated anti-CD126 (IL-6R), and PC5-conjugated anti-CD14 to identify monocytes.

### 2.3. Procedures

After explaining the details of the study and screening potential participants for inclusion and exclusion criteria, an assessment appointment was made. Participants who reported having persistent painful peripheral neuropathy that started after the initiation of chemotherapy composed the CIPN group (*N* = 20) while those who reported having no symptoms of peripheral neuropathy during or after chemotherapy made up the comparison group (*N* = 20). Recruitment continued until there were 20 participants in each group. 

All assessments were scheduled within the same 2-hour time period in the morning (8–10 AM) to control for diurnal variations in immune parameters. Participants were asked to refrain from consuming food, drinking alcohol and/or caffeine, using tobacco, taking nonprescription medication, and engaging in strenuous exercise during the 12-hour period before their appointment. At the appointment, a sensory and motor exam was conducted by a clinician trained in neurosensory testing in order to classify the grade of CIPN or verify the absence of symptoms. Participants were then asked to complete the study questionnaires and have their blood drawn (20 ml) for analysis of IL-6 and IL-6 receptors. Blood was drawn using a standard venipuncture protocol and collected into one serum separator vacutainer tube, and one heparinized vacutainer tube. After the blood draw, the vacutainers were placed on ice and transported directly to the laboratory for processing.

#### 2.3.1. Data Analysis and Interpretation

All data was entered into SPSS version 16.0 (Chicago, IL). Shapiro-Wilk's test of normality was performed. The cytokine data, which was not normally distributed, were transformed logarithmically and retested to ensure normal distribution before analysis by univariate analysis of variance (ANOVA). Further analysis using the Levene test showed equality of variances between groups after logarithmic transformation. The primary aim of the study, to examine the levels of IL-6 and IL-6 receptors (IL-6R and sIL-6R) in women who reported either painful CIPN symptoms or no CIPN symptoms following BCA treatment, was analyzed using independent *t*-tests with Bonferroni corrections set at the 0.05 level of significance. Analyses of covariance controlled for possible confounders in comparisons between the painful CIPN and Comparison group. Covariates included age, body mass index, time postdiagnosis, and treatment modality. The secondary aim, to explore the relationships among biological factors and QOL, was analyzed using Pearson correlation coefficients with significance set at the 0.05 level (2-tailed).

## 3. Results

The study sample characteristics can be found in Table 1. Both groups were composed of Caucasian, non-Hispanic participants, with grade II-III BCA who had surgical tumor resection of the breast with subsequent chemotherapy treatment. The mean time since diagnosis for the CIPN group was 8.2 months and 9.1 months for the Comparison group. The mean body mass index (BMI) of the CIPN group was 29.8 (±5.2) and 28.4 (±4.9) in the Comparison group. All participants received a combination chemotherapeutic regimen. There were no significant differences between groups in terms of age, education, income level, BMI, or comorbidities. The most common comorbidities were hypertension, osteoporosis, and seasonal allergies. 

All CIPN participants were classified as grade 2 or 3 using the National Cancer Institute (NCI) Toxicity Criteria. The CIPN participants rated their pain using the MPQ-SF. The sensory aspects of pain had a mean score of 9 (±3) with “hot burning” being the most common description selected. Affective components of pain had a mean score of 3 (±1.3) with “tiring-exhausting” being selected most often. The total pain rating index had a mean of 11.6 (±4.3) with a present pain intensity score of 57 (±14) out of 100. The evaluative overall intensity of total pain experience had a mean score of 2 (±1.0), reflecting the perception of the pain experience as “discomforting”. 

**Figure 1:**
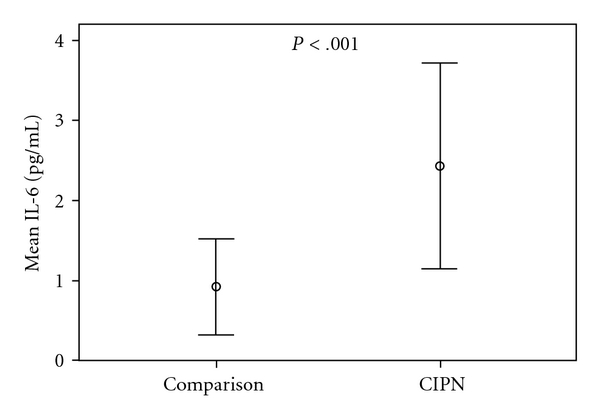
Mean levels of serum interleukin [IL]-6 in women with painful CIPN (*N* = 20) and women without CIPN symptoms (Comparison Group, *N* = 20). Dots represent mean value of Interleukin [IL]-6 measured in serum; error bars represent 95% CI. Mean (SD), [Range] of the painful CIPN group: 2.43 (1.7) pg/mL [1.3–3.8]; Comparison group: 0.92 (0.6) pg/mL [0.4–1.5]. Independent *t*-test with Bonferroni correction performed to examine difference between groups: (*t*(38) = −5.6, *P* < .001).

**Figure 2:**
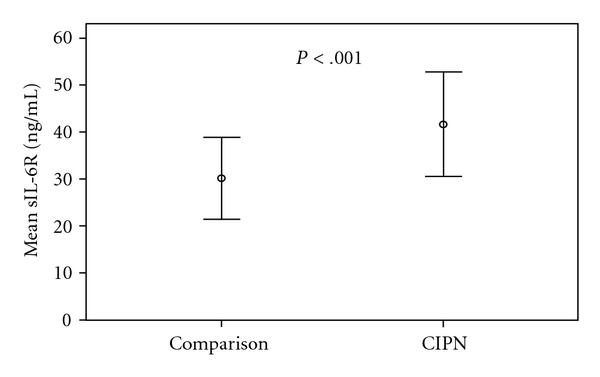
Mean levels of serum Soluble IL-6R in women with painful CIPN (*N* = 20) and women without CIPN symptoms (Comparison Group, *N* = 20). Dots represent mean value of soluble IL-6R (sIL-6R) measured in serum; error bars represent 95% CI. Mean (SD), [Range] of the Painful CIPN group: 41.6 (9.7) ng/mL [31–54]; Comparison group: 30.2 (5.8) ng/mL [22–39]. Independent *t*-test with Bonferroni correction performed to examine difference between groups: (*t*(38) = −5.1, *P* < .001).

**Figure 3:**
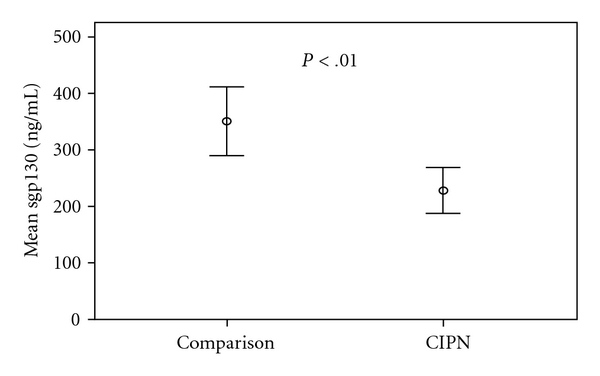
Mean levels of serum soluble gp130 in women with painful CIPN (*N* = 20) and women without CIPN symptoms (Comparison Group, *N* = 20). Dots represent mean value of soluble gp130 (sgp130) measured in serum; error bars represent 95% CI. Mean (SD), [Range] of the Painful CIPN group: 228.3 (112.7) ng/mL [198–263]; Comparison group: 350.8 (119.9)  ng/mL [290–407]. Independent *t*-test with Bonferroni correction performed to examine difference between groups: (*t*(38) = 4.2, *P* < .01).

Interleukin-6 (Figure 1) and soluble IL-6R (sIL-6R, Figure 2) levels were significantly higher in the painful CIPN group compared to women without CIPN symptoms (comparison group) (IL-6, 2.43 versus 92 pg/mL; *t*(38) = −5.6, *P* < .001; sIL-6R,41.6 versus 30.2 ng/mL; *t*(38) = −5.1, *P* < .001, resp.). In addition, soluble gp130 (Figure 3), which blocks the IL-6/sIL-6R complex from binding to gp130 within the cell membrane, was significantly lower in the CIPN group compared to women without CIPN symptoms (sgp130, 228.3 versus 350.8 ng/mL; *t*(38) = 4.2, *P* < .01). Relationships among pain and the biological factors are shown in Table 2.

The CIPN group had significantly lower levels of IL-6R on CD14^+^ cells (CD126/CD14 percentage of positive cells: 29% versus 42%; *t*(38) = −2.26, *P* = 0.03). Differences in these biomarker concentrations remained significant after controlling for age, BMI, number of comorbidities, and time postdiagnosis in an analysis of covariance. Elevated levels of sIL-6R can cause cytokine-induced receptor shedding, leading to decreased cell-surface expression of IL-6R. Consistent with this, monocyte cell-surface expression of IL-6R was negatively correlated with circulating levels of sIL-6R in the total sample (*r* = −.614, *P* = .005).

The CIPN participants scored significantly lower on all subscales of the SF-36 Health Survey except the “role emotional” subscale compared to the women without CIPN symptoms (data not shown; all *Ps* < .002). Significant relationships were identified between the biological measures and QOL subscales as shown in Table 3. Levels of IL-6 and sIL-6R were negatively correlated with general health, physical functioning, role functioning, social functioning, bodily pain, vitality, and mental health. Higher levels of pain are reflected as lower scores on the SF-36 bodily pain subscale. Levels of sgp130 were positively correlated with most QOL subscales. 

## 4. Discussion

The BCA participants with painful CIPN in this study had significantly higher levels of circulating IL-6 and sIL-6R than participants without CIPN (Comparison group). Consistent with a potential inflammatory basis for painful CIPN symptoms via increased IL-6 transsignaling, elevated levels of sIL-6R were accompanied by a significant reduction in monocyte cell-surface expression of IL-6R. The density of IL-6R on the cell-surface of monocytes was also inversely correlated with circulating levels of sIL-6R in the total sample (*r* = −.614, *P* = .005). Furthermore, IL-6 and sIL-6R levels were inversely correlated with most health-related QOL categories. These findings suggest that painful CIPN symptoms and reduced QOL are linked with activation of gp130 via the IL-6/sIL-6R complex as opposed to the more restricted mechanism of IL-6 binding directly to its membrane-bound receptor, IL-6R [33]. 

Rencently, Andratsch et al. [34] reported that knock-out mice lacking gp130 selectively in nociceptive neurons of the dorsal root ganglion did not show behaviors consistent with inflammatory-induced hyperalgesia when exposed to noxious heat. Their findings indicate that gp130 expressed in nociceptive primary afferents required for the development of pathological pain and hyperalgesia. Increased levels of IL-6 and sIL-6R may therefore increase the risk of experiencing CIPN through activation of gp130 on sensory nerves. 

When the peripheral nerve is damaged, immune-modulating cells, such as lymphocytes and macrophages, release proinflammatory cytokines in order to home other immune cells to the area of injury. These cells then infiltrate through the blood-nerve barrier, exposing the injured tissue to a host of inflammatory mediators [35]. Kiguchi and colleagues [36] recently demonstrated that repeated vincristine administration causes an increase in invading peripheral macrophage-derived IL-6 expression.When a neutralizing antibody of IL-6 was injected into the area surrounding the sciatic nerve, it suppressed the development of vincristine-induced mechanical allodynia. Thus, peripheral IL-6 may be an important factor involved in the pathophysiological changes that cause painful CIPN symptoms. Increased levels of infiltrating macrophages were also observed after administration of paclitaxel [37], which was subsequently linked with increased expression of matrix metalloproteinase (MMP)-3, another factor influenced by proinflammatory cytokine concentrations [38].

Additional cytokines may also be involved in the cascade of events leading to painful CIPN. A recent study investigated mRNA levels of proinflammatory cytokines interleukin-2 (IL-2) and tumor necrosis factor-alpha (TNF) as well as anti-inflammatory cytokines IL-4 and IL-10 among patients with painful neuropathy, nonpainful neuropathy and control participants [12]. Patients with painful neuropathy had a two-fold increase in IL-2 and TNF, while patients with nonpainful neuropathy had significantly higher mRNA levels of IL-4 and IL-10. This study supports the hypothesis that interactions between pro- and anti-inflammatory cytokines are important for understanding the pathophysiological changes associated with pain. While it is unlikely that IL-6 activity is the only factor determining the presence of painful CIPN symptoms, it may predispose individuals to the biological interactions that result in the release of pain-inducing substances. Clearly, to determine the significance of IL-6 activity and other cytokine interactions on painful CIPN symptoms, further research is needed to characterize a broad spectrum of immune factors over the treatment trajectory. Studies that are able to control for the type of chemotherapeutic agent administered are especially needed, as it is possible that the induction of various mechanisms that lead to elevated levels of proinflammatory cytokines may account for the variability in CIPN symptoms and health-related QOL [39].

In the present study, participants with painful CIPN had significantly lower QOL scores compared to the Comparison group. This finding is consistent with past research [40]. A recent study conducted among 6000 adults compared QOL among people with and without chronic pain [41]. The pain group was subdivided into a chronic neuropathic pain group (*N* = 241) and a chronic pain group (*N* = 1179). Chronic neuropathic pain was associated with severely reduced functioning in all aspects of daily activities and on every dimension of QOL measured by the SF-36 Health Survey. Even after adjusting for age and severity of pain, the health and daily activity in chronic neuropathic pain was poorer as compared to the chronic pain group or no-pain group. In the present study, IL-6 and sIL-6R were negatively correlated with most QOL subcategories, suggesting that increased IL-6 activity is involved in CIPN-related QOL impairments.

The results of the present study provide some evidence that IL-6 and sIL-6R may be potential therapeutic targets for painful CIPN. Clinical trials using neutralizing anti-IL-6R antibody in patients with rheumatoid arthritis and inflammatory pain have been promising, with significant reductions in pain perception and other inflammatory-related symptoms in patients receiving anti-IL-6R antibody [42, 43]. Notably, these agents work by inhibiting IL-6R directly while preserving levels of sgp130. While more research is needed to determine whether these agents will be effective in ameliorating CIPN symptoms, it provides hope that a treatment may be found.

Nurses providing care to patients who are receiving chemotherapeutic agents and other immune-modulating medications can play a key role in recognizing potential cytokine-related symptoms. The presence of cooccurring symptoms (pain, fatigue, depression) associated with proinflammatory cytokines has been reported [10, 33]. Nursing research focused on identifying patients at risk of experiencing painful CIPN and reduced QOL may wish to consider including other symptoms, such as depression and fatigue, which are known to be influenced by cytokine concentrations, particularly IL-6. Ultimately, a deeper understanding of the biological interactions that influence symptoms of disease and/or treatment is needed to enable the development of strategies to identify patient at risk of poor outcomes and effective nursing interventions that will improve symptom management and QOL in women with BCA.

### 4.1. Limitations

 This study had several limitations that need to be discussed, most notable being the small sample size which included an all white, non-Hispanic population, and the cross-sectional design with only one measurement time-point. Although the main goal of the study was to examine the concentrations of IL-6 and IL-6 receptors across BCA-treatment groups, the differences in chemotherapeutic protocols or other adjunctive treatment may have affected these results. For instance, it was recently reported that women receiving taxanes were more than twice as likely to develop CIPN, whereas women receiving platinum-taxane combination therapy were more than three times as likely to develop CIPN compared to women who did not receive chemotherapy [44]. In addition, measurement of other acute phase proteins, such as c-reactive protein (CRP), would have strengthened the study by providing a quantitative functional evaluation of the resulting IL-6 activity. Since CRP measurement was not a part of the original study design, financial constraints prohibited its inclusion. 

The study may also be criticized for the analysis of cell-surface IL-6R, which was restricted to a specific cell type (monocytes) instead of including neutrophils or lymphocytes. Monocyte cell-surface IL-6R was selected for this study because this cell type exhibits a sustained response after immune activation, the mean time of CIPN symptoms was 8.2–9.1 months, and alterations in the number of monocyte IL-6R receptors has been associated with other negative symptoms in BCA populations [33]. Although cytokine measurements and flow cytometry data were performed on serum and leukocytes purified from blood representing overall IL-6 production and secretion from all cell types, the major source of soluble IL-6R in persistent inflammation is from monocytes [12, 25] whereas neutrophils and lymphocytes are primary sources during the acute inflammatory response [27]. As a plausible cause of persistent CIPN symptoms, chronic inflammation in this patient population was evaluated by changes in IL-6 activity through the loss of cell-surface IL-6 on monocytes, but this does not infer that neutrophils and lymphocytes are not involved. Future research may consider including cell-surface IL-6R on all leukocyte cell types so that the role of acute versus chronic inflammatory activation on CIPN outcomes may be better understood.

All participants in the study were evaluated by an experienced clinician trained in neurosensory testing and were graded for symptoms of CIPN according to the NCI Toxicity Criteria. However, other measures for subjectively rating CIPN symptoms are now available, which may be more accurate in determining the effect of CIPN on functioning compared to a clinician's assessment [45, 46]. The use of these instruments should be considered both in the clinical setting and in future CIPN research. 

In addition, because this study was exploratory in nature, no cause-effect relationship can be implied. Thus, although elevated IL-6 activity was found among participants with painful CIPN, it remains a potential mechanism involved in the development of painful CIPN symptoms. However, noting the accumulating evidence in pain research concerning the role of cytokines in immune activation and the resultant cascade of events that lead to pain, the role of increased IL-6 and soluble IL-6 receptors in women with persistent painful CIPN and reduced health-related QOL warrants further consideration.

## 5. Conclusions

Women with painful CIPN had significantly higher levels of IL-6 and sIL-6R, and significantly lower levels of sgp130 and monocyte cell-surface IL-6R than women without symptoms of CIPN after BCA treatment. Although no causal relationship can be implied, the results of this study suggest an inflammatory basis for painful CIPN symptoms among women with BCA, possibly through increased binding of IL-6/sIL-6R with subsequent activation of gp130. With evidence accumulating about the role of immune activation in causing neuropathic pain, IL-6 transsignaling, as well as other cytokine interactions warrants further attention in CIPN research. 

The biological measures, IL-6 and sIL-6R, were negatively correlated with most QOL subscales on the SF-36 Health Survey whereas sgp130 was positively correlated with most subscales. These findings imply that as IL-6 increases its capability to activate gp130 via IL-6 transsignaling, health-related QOL declines. With a potential influence on physical, social, and emotional functioning, symptom researchers may wish to consider evaluating the influence of IL-6 activity on cooccuring symptoms in cancer.

The findings of this study inform nurses that persistent CIPN symptoms and reduced QOL may be related to the inflammatory mediator, IL-6, and increased levels of its soluble receptor. Diligent assessment of neurological functioning and QOL over the disease trajectory and in survivorship is required to identify and assist those at risk of experiencing persistent symptoms. The results also support the need to examine cytokine interactions throughout the treatment trajectory in women with BCA in order to determine the effect on painful CIPN, QOL, and possibly other co-occurring symptoms. Longitudinal immune data categorized by the type of chemotherapeutic agent(s) administered may provide valuable information on the interactions that lead to painful CIPN and reduced health-related QOL, as well as factors which predispose individuals to experiencing persistent symptoms in survivorship.

## Figures and Tables

**Table 1:** Sample Characteristics.

Demographic variable	Chemotherapy-induced peripheral neuropathy group (*N* = 20)		Comparison group (*N* = 20)	
Age [mean ± SD(range)]	56.8 + 6.6 years (42–65)		53.4 ± 5.6 years (43–65)	
Marital status	Married	12 (60%)	Married	11(55%)
	Divorced	5 (25%)	Divorced	7 (35%)
	Widowed	3 (15%)	Widowed	2 (10%)
Education	High school	11 (55%)	High school	10 (50%)
	College	5 (25%)	College	7 (35%)
	Postgraduate	4 (20%)	Postgraduate	3 (15%)
Income level	<$50 K/year	6 (30%)	<$50 K/year	7 (35%)
	$50–74 K/year	6 (30%)	$50–74 K/year	6 (30%)
	>$75 K/year	8 (40%)	>$75 K/year	7 (35%)
Tobacco Use	Never	16 (80%)	Never	15 (75%)
	Current or past use	4 (20%)	Current or past use	5 (25%)
Comorbid conditions	0 conditions	7 (35%)	0 conditions	8 (40%)
	1 condition	8 (40%)	1 condition	10 (50%)
	2 conditions	4 (20%)	2 conditions	1 (5%)
	3 or more conditions	1 (5%)	3 or more conditions	1 (5%)

Independent *t*-tests were used to analyze demographic data. There were no significant differences between the painful CIPN and Comparison groups on any of the demographic or treatment-related variables.

**Table 2:** Pearson correlation coefficients among biological measures and pain perception.

	IL-6 (pg/mL)	sIL-6R (ng/mL)	sgp130 (ng/mL)
Pain (total rating index)	.519**	.762**	−.825**
IL-6 (pg/mL)	1	.613**	−.801**
sIL-6R (ng/mL)	.613**	1	−.692**
sgp130 (ng/mL)	−.801**	−.692**	1

**P < .01.

**Table 3:** Pearson correlation coefficients among biological measures and quality of life.

	GH	PF	RF	RE	SF	BP	V	MH
IL-6 (pg/mL)	−.614**	−.724**	−.686**	−.062	−.374*	−.815**	−.509**	−.418**
sIL-6R (ng/mL)	−.551**	−.526**	−.531**	−.136	−.386*	−.687**	−.358*	−.419**
sgp130 (ng/mL)	.796**	.733**	.782**	.124	.478**	.850**	.511**	.503**

SF-36 Health Survey Subcategories; GH: general health; PF: physical functioning; RF: role functioning; RE: role emotional; SF: social functioning; V: vitality; MH: mental health; *P < .05, **P < .01.
